# Sensor Fault Diagnosis for Impedance Monitoring Using a Piezoelectric-Based Smart Interface Technique

**DOI:** 10.3390/s20020510

**Published:** 2020-01-16

**Authors:** Thanh-Canh Huynh, The-Duong Nguyen, Duc-Duy Ho, Ngoc-Loi Dang, Jeong-Tae Kim

**Affiliations:** 1 Faculty of Civil Engineering, Duy Tan University, 03 Quang Trung, Hai Chau, Danang 550000, Vietnam; huynhthanhcanh@duytan.edu.vn (T.-C.H.); theduong.nguyen@duytan.edu.vn (T.-D.N.); 2 Center for Construction, Mechanics and Materials, Institute of Research and Development, Duy Tan University, 03 Quang Trung, Hai Chau, Danang 550000, Vietnam; 3 Faculty of Civil Engineering, Ho Chi Minh City University of Technology (HCMUT), VNU-HCM, 268 Ly Thuong Kiet, District 10, Ho Chi Minh City 700000, Vietnam; hoducduy@hcmut.edu.vn or; 4 Ocean Engineering Department, Pukyong National University, 45 Yongso-ro, Daeyeon 3-dong, Namgu, Busan 48513, Korea; loi.ngocdang@gmail.com

**Keywords:** piezoelectric sensor, smart interface, sensor diagnosis, breakage, debonding, shear-lag effect, impedance method, damage detection, electromechanical impedance

## Abstract

For a structural health monitoring (SHM) system, the operational functionality of sensors is critical for successful implementation of a damage identification process. This study presents experimental and analytical investigations on sensor fault diagnosis for impedance-based SHM using the piezoelectric interface technique. Firstly, the piezoelectric interface-based impedance monitoring is experimentally conducted on a steel bolted connection to investigate the effect of structural damage and sensor defect on electromechanical (EM) impedance responses. Based on the experimental analysis, sensor diagnostic approaches using EM impedance features are designed to distinguish the sensor defect from the structural damage. Next, a novel impedance model of the piezoelectric interface-driven system is proposed for the analytical investigation of sensor fault diagnosis. Various parameters are introduced into the EM impedance formulation to model the effect of shear-lag phenomenon, sensor breakage, sensor debonding, and structural damage. Finally, the proposed impedance model is used to analytically estimate the change in EM impedance responses induced by the structural damage and the sensor defect. The analytical results are found to be consistent with experimental observations, thus evidencing the feasibility of the novel impedance model for sensor diagnosis and structural integrity assessment. The study is expected to provide theoretical and experimental foundations for impedance monitoring practices, using the piezoelectric interface technique, with the existence of sensor faults.

## 1. Introduction

The electromechanical (EM) impedance method has been extensively investigated for structural integrity assessment of critical connections in civil structures [1,2,3,4,5,6]. The method relies on acquiring high-frequency impedance responses which are sensitive to incipient structural damages [7,8,9]. Traditionally, a piezoelectric sensor (e.g., PZT) is bonded directly to the host structure’s surface to perform a required impedance measurement. To detect structural damage, the measured impedance signature is statistically compared with the signature of the undamaged state by using statistical damage metrics [10,11,12]. However, the direct attachment of the PZT often leads to weak EM impedance responses and further results in difficulties in predetermining effective frequency bands for damage detection tasks [13,14,15]. To overcome these issues, the piezoelectric-based smart interface technique (i.e., the PZT interface) has been developed as an alternative measurement for the PZT sensor [5,16,17,18]. The PZT is indirectly attached to the host structure via a substrate member called ‘interface’. The structural and geometrical properties of the PZT interface are adjusted to create strong resonances in a desired effective frequency range [19]. The piezoelectric interface technique can be easily integrated with a wireless impedance sensing system to perform an autonomous and real-time structural health monitoring (SHM) [20,21,22].

However, challenges still exist against the in-situ application of the piezoelectric-based smart interface technique. To ensure the success of a damage identification process, it is critical to secure the operational condition of the piezoelectric sensor. In reality, overloading conditions, material deteriorations, and environmental changes acting on the monitored structure could inevitably lead to the degradation of the sensor and its bonding layer. The sensor breakage or quality degradation would cause the change in piezoelectric properties. In addition, the bonding layer’s defect would affect the force transmission from the PZT to the interface structure (via the shear mechanism) [23]. These will result in observable changes in measured EM impedance responses, which would be falsely interpreted as the existence of structural damages.

Many research efforts have been made to detect the fault of sensors in SHM systems [24,25,26,27]. While most of them have focused on the vibration method, only a few studies have evaluated the operational condition of piezoelectric sensors for the impedance method. Giurgiutiu et al. [28] studied the influence of the bonding layer on the EM impedance and proposed a sensor debonding identification method by tracking the appearance of the PZT’s resonance. Park et al. [29] developed a sensor self-diagnostic strategy tailored to the impedance method by tracking the changes in the gradients of imaginary admittance (i.e., an inverse of impedance). Ai et al. [30] extracted the features of the real admittance responses and used them to diagnose and differentiate the sensor faults from the structural damage. Despite those research attempts, the diagnosis of sensor defects has not been extensively investigated for the piezoelectric interface-based impedance monitoring. Thus, there exists a need to experimentally examine the sensor self-diagnosis feature and to assess the sensor defects for the piezoelectric interface technique.

In the past few decades, several simplified impedance models have been developed to analytically interpret the self-diagnosis feature of piezoelectric sensors. On the basis of Liang’s model [31], Xu and Liu [32] proposed an analytical model which can be used to estimate the debonding influence of the adhesive layer on the EM impedance. Bhalla and Soh [23] formulated a shear-lag model to analyze the mechanism of force transfer through the bonding layer between the PZT and the host structure. The shear-lag model was then upgraded by including the shear stress and the inertial in the EM impedance derivation [33]. Park et al. [34] extended the impedance model of Xu and Liu [32] to take into account the degradations in the sensor quality and the bonding layer. Recently, 2-degree-of-freedom (2-dof) impedance models have been proposed to analytically represent the coupled interaction between the piezoelectric-based smart interface and the host structure [20,35]. Nonetheless, the 2-dof impedance models as they have been developed so far can be only used to estimate the structural integrity because they have ignored the role of the bonding layer and idealized the force transfer between the PZT and the interface. Therefore, a more complete and rigorous impedance model, which can be used for both structural integrity and sensor defect assessments, should be sought for the smart interface technique.

In this study, comprehensive experimental and analytical investigations on the diagnosis of sensor fault are presented for impedance-based SHM using the smart interface technique. For the experimental investigation, impedance measurements on a bolted connection are conducted to analyze the influences of sensor defects and structural damages on the EM impedance responses. Based on the experimental observation, sensor self-diagnostic methods using impedance features are outlined to detect and discriminate the sensor defect (i.e., breakage and debonding) from the occurrence of structural damage. In the analytical investigation, a novel impedance model of the PZT interface including the bonding layer is formulated for a mechanistic interpretation of the effect of the sensor fault. Various parameters are introduced into the EM impedance formulation to represent shear-lag phenomenon, sensor defect, and structural damage. Lastly, a parametric study is performed by using the analytical impedance model to estimate the change in EM impedance according to various sensor faults and structural damages. The analytical EM impedance modeling is validated by comparing the analytical results with the experimental observations.

## 2. Experimental Investigation

### 2.1. Test on Bolted Joint

#### 2.1.1. Test-Setup

**Figure 1:**
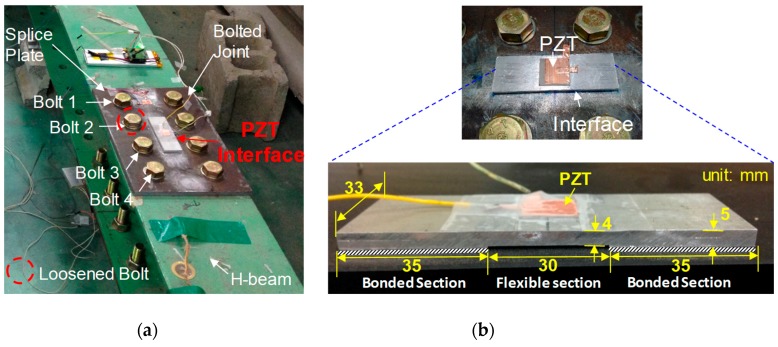
Experimental setup of the lab-scaled steel beam: (**a**) setup of bolted joint; (**b**) prototype of PZT interface.

The bolted connection of a lab-scaled steel beam was selected to investigate the effect of sensor defects (i.e., sensor breakage and debonding) on impedance monitoring via the PZT interface device. The theoretical background of the PZT interface-based impedance monitoring technique for damage monitoring of bolted joints is reported in [20]. As shown in Figure 1a, the steel beam has an H-shaped cross-section with the dimensions of H−200 × 180 × 8 × 10 mm. The beam connection was fastened by 8 bolts (ϕ20 mm) at the top and the bottom flanges, respectively. The splice connection has a wide of 310 mm, a height of 200 mm, and a thickness of 10 mm. All bolts were fastened to a torque level of 160 Nm to simulate the initial condition of the joint (i.e., a healthy state).

To acquire the EM impedance from the joint, a PZT interface was fabricated and mounted to the surface of the splice plate as shown in Figure 1a. The interface is an aluminum plate-like member that has three sections: two outside bonded sections and a middle flexible section, as sketched in Figure 1b. A PZT element (PZT-5A) was bonded to the flexible section via a bonding layer formed by an instant adhesive layer (Loctite 401). The flexible section was designed to provide a free vibration for the PZT element during the piezoelectric excitation. As detailed in Figure 1b, the flexible section has a width of 33 mm, a length of 30 mm, and a thickness of 4 mm; and the bonded sections have a width of 33 mm, a length of 30 mm, and a thickness of 5 mm. The PZT sensor has a width of 25 mm, a height of 25 mm, and a thickness of 0.51 mm. For impedance measurement, the HIOKI 3532 analyzer (HIOKI E.E. Corporation, Japan) was used to generate harmonic excitations of 1 V and to measure EM impedance responses. The detailed design of the piezoelectric interface prototype for the bolted joint can be found in an existing publication [20].

#### 2.1.2. Test Scenario

**Figure 2:**
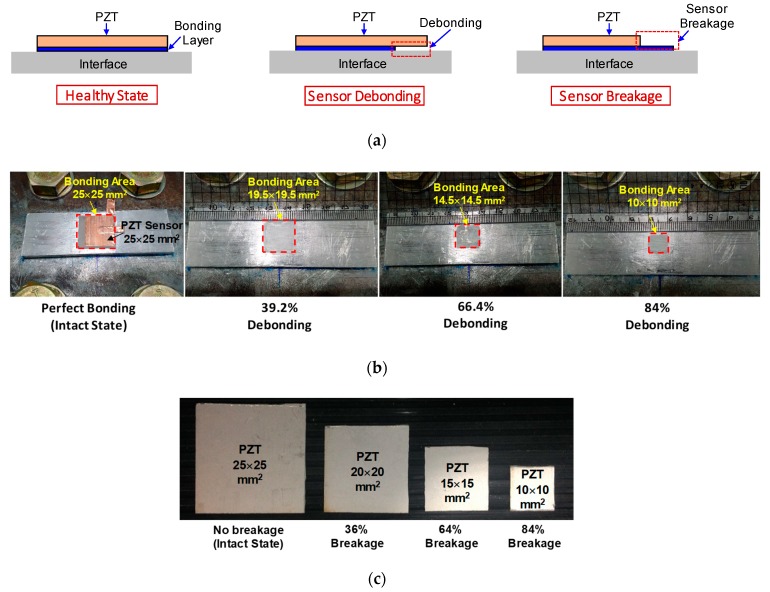
Ideal simulation of sensor debonding and sensor breakage: (**a**) illustration of sensor defects; (**b**) sensor debonding cases; (**c**) sensor breakage cases.

The experimental investigation was carried out for three different patterns including sensor debonding, sensor breakage, and structural damage. Due to the difficulty of forming realistic sensor debonding and breakage conditions, simple ideal simulations were made as shown in Figure 2. Firstly, the test on sensor debonding was performed by reducing the bonding area of the adhesive layer, as shown in Figure 2a. Accordingly, four cases of sensor debonding were investigated as follows: no debonding (i.e., an intact state), 39.2% debonding, 66.4% debonding, and 84% debonding, as illustrated in Figure 2b. Secondly, the test on sensor breakage was simulated by reducing the size of the PZT, see Figure 2a. Four cases of PZT sensor breakage were then studied as follows: no breakage (i.e., an intact state), 36% breakage, 64% breakage, and 84% breakage, as shown in Figure 2c. Thirdly, the test on structural damage was conducted by reducing the torque of Bolt 2, as indicated in Figure 1a. Accordingly, the torque of Bolt 2 was reduced from 160 Nm to 110 Nm, 60 Nm, and 0, respectively, to simulate the structural damage.

The bonding condition of the PZT sensor mounted on the interface was controlled during the curing of the adhesive layer. The bonding condition was visually checked to scale the bonding area. It is reported that the temperature has considerable effects on the impedance response of the smart interface [36,37]. The baseline of the impedance signature varies with the temperature change. To accurately quantify the effect of sensor defects on the impedance, the room temperature was controlled at 21 °C during the experiment.

### 2.2. Effect of Sensor Defect on Impedance Response

**Figure 3:**
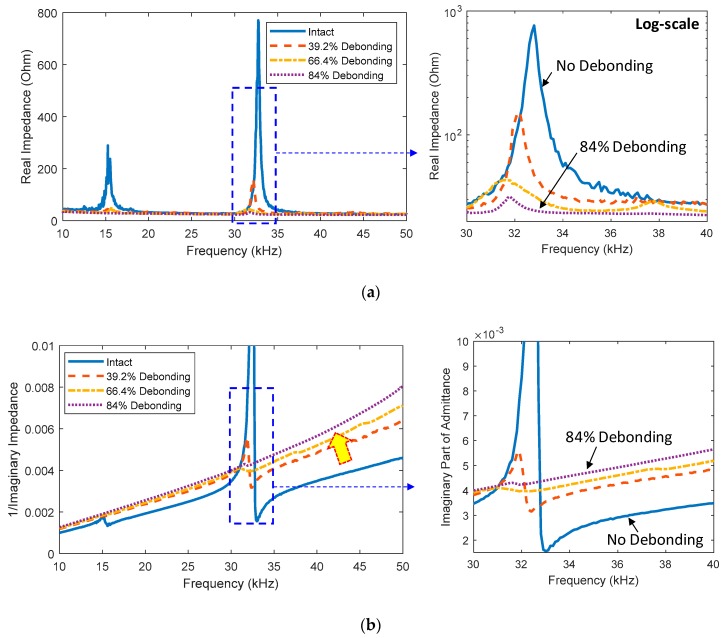
Measured impedance signatures under the sensor debonding cases: (**a**) real part; (**b**) imaginary part.

For the sensor debonding cases, the real parts of impedance signatures were measured in the frequency range of 10–50 kHz as shown in Figure 3a. The signatures show two clear resonant peaks under the intact state of the PZT (i.e., a perfect bonding condition). When the debonding level was increased up to 84%, the magnitude of the resonant impedance peaks was reduced, as zoomed in on in Figure 3a. It is known that the imaginary part of impedance contains much information about the sensor’s health status. Hence, the effect of the sensor debonding on the imaginary admittance (i.e., an inverse of impedance) was examined in Figure 3b. It is observed that the sensor debonding caused the upward shift in the slope of the imaginary admittance.

**Figure 4:**
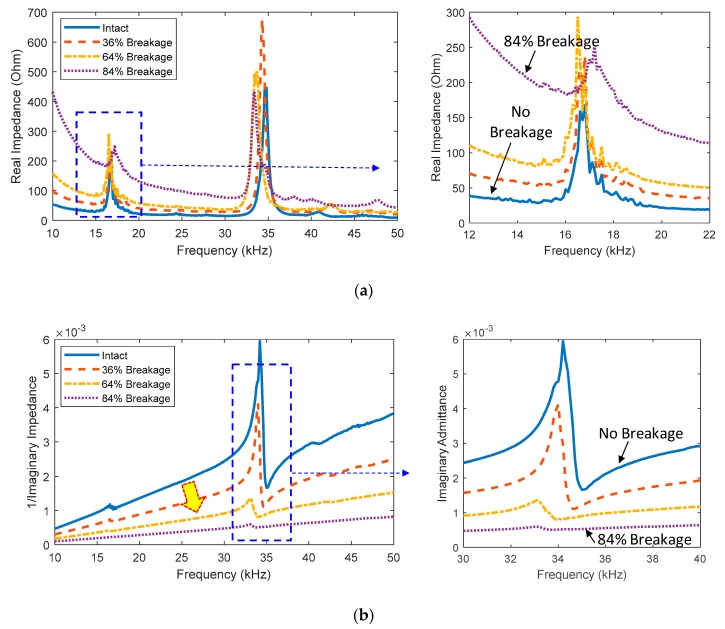
Measured impedance signatures under three cases of sensor breakage: (**a**) real part; (**b**) imaginary part.

Figure 4a shows the real impedance signatures in 10–50 kHz for different levels of the sensor breakage. Obviously, the sensor breakage caused upward shifts in the real impedance signatures, as zoomed in on in Figure 4a. The shifting effect was found to be more considerable for lower frequencies. The imaginary admittance signatures in 10–50 kHz were also examined for the different breakage levels of the PZT, as shown in Figure 4b. It is obvious that the sensor breakage caused downward shifts in the slope of the imaginary admittance, as zoomed in on in Figure 4b.

**Figure 5:**
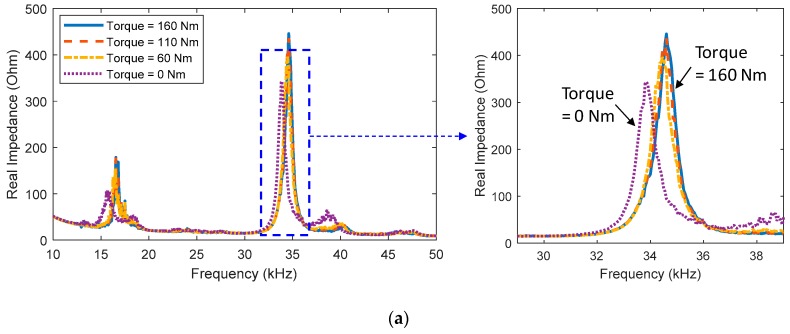

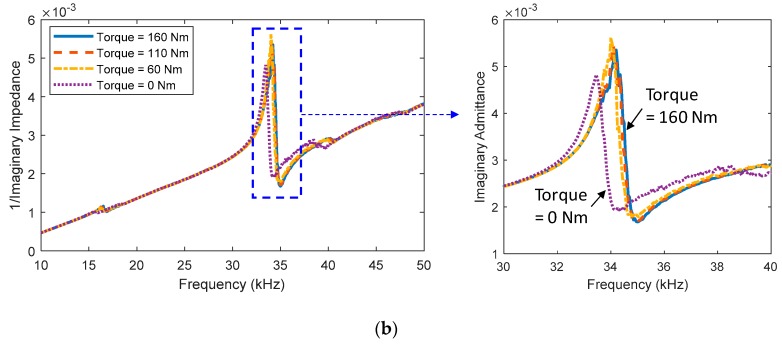
Measured impedance signatures under the structural damage cases (Bolt 2 loosened): (**a**) real part; (**b**) imaginary part.

For the structural damage cases, the real impedance signatures in 10–50 kHz were shown in Figure 5a. The reduction in bolt torque caused leftward shifts in the real impedance at the resonances. The torque reduction also significantly modified the imaginary admittance signatures at the resonances, as observed in Figure 5b. The structural damage slightly causes small shifts in the slope of the imaginary admittance.

The effect of the sensor fault and the structural damage on the EM impedance was quantified by root-mean-square-deviation (RMSD). The RMSD feature statistically measures the difference between two impedance signatures according to the following formulas:

(1) $$\mathrm{RMSD}=\sqrt{\sum_{i=1}^{N}{\left[Z^{*}(\omega_{i})-Z(\omega_{i})\right]}^{2}/\sum_{i=1}^{N}{\left[Z(\omega_{i})\right]}^{2}},$$

where *Z*(*ω_i_*) and Z^*^(*ω_i_*) signify the baseline impedance and the current impedance at the *i*th frequency, respectively; $\bar{Z}$ and ${\bar{Z}}^{*}$ signify the mean values of the baseline and current impedance signatures; $\sigma_{Z}$ and $\sigma_{Z}^{*}$ are the corresponding standard deviations; *N* is the number of swept frequencies.

**Figure 6:**
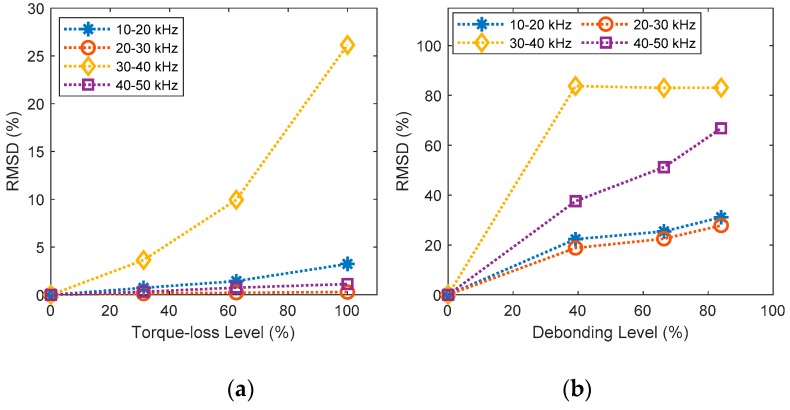

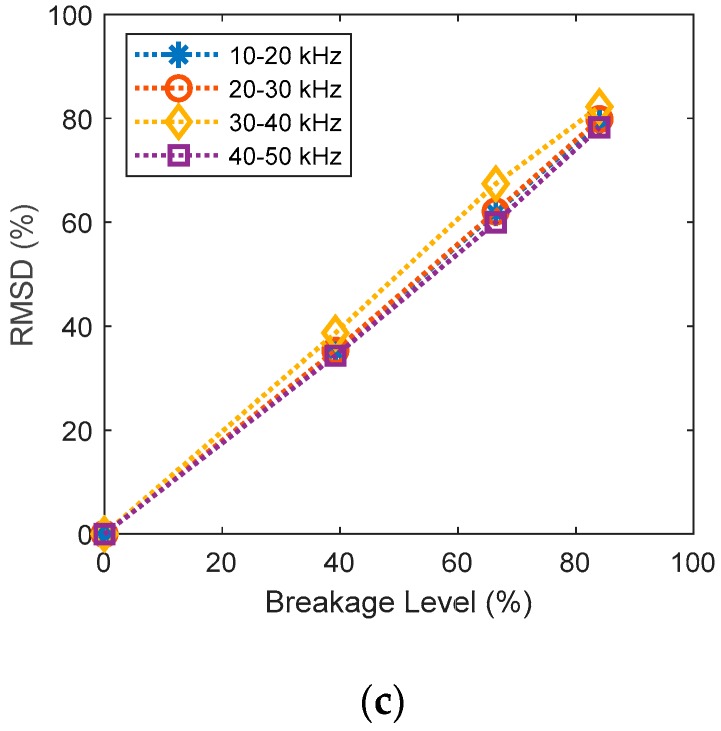
The RMSD metric of imaginary impedance signatures: (**a**) structural damage; (**b**) sensor debonding; (**c**) sensor breakage.

The RMSD feature was computed for resonant frequency zones (i.e., 10–20 kHz and 30–40 kHz) and non-resonant frequency zones (i.e., 20–30 kHz and 40–50 kHz) of the imaginary impedance signatures. As shown in Figure 6a, the resonant frequency range (30–40kHz) showed the significant change under the structural damage while the non-resonant frequency ranges showed only ignorable changes under the structural damage cases. This is because the non-resonant ranges contain less information about the structural damage than the resonant ranges. As shown in Figure 6b,c, all frequency ranges (i.e., non-resonant and resonant ranges) showed the significant change under the sensor debonding and breakage. It is confirmed that all examined frequency ranges of the imaginary impedance contain rich information about the integrity condition of the sensor and bonding layer.

### 2.3. Sensor Self-Diagnosis Using Impedance Features

**Figure 7:**
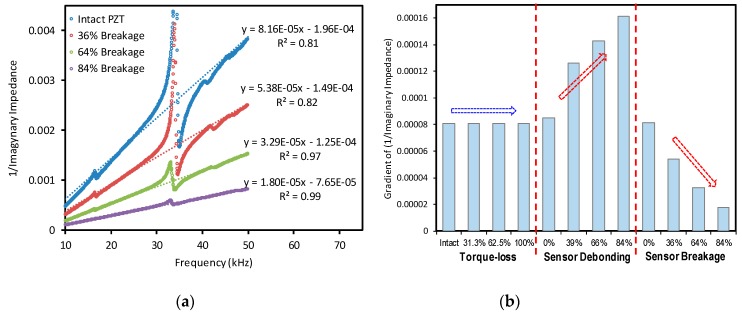
Sensor fault diagnosis using the slope of imaginary admittance: (**a**) linear approximation; (**b**) sensor fault diagnosis.

Two impedance features were examined to distinguish the sensor defect from the structural damage of the bolted joint. At first, the slopes of the imaginary admittance signatures were computed by using the linear approximation method, as shown in Figure 7a. The computed slopes were then plotted according to the severities of the structural damage (i.e., bolt’s torque-loss), the sensor debonding, and the sensor breakage, as depicted in Figure 7b. The results showed that the slope of the imaginary impedance remained generally constant about 8 × 10^−5^ for the structural damage cases. When the sensor was debonded, the slope of the imaginary admittance was rapidly increased. Particularly, the slope was changed from 8 × 10-5 to 16 × 10^−5^ as the debonding severity rose from 0% to 84%. For the sensor breakage cases, the slope was decreased from 8 × 10^−5^ to 1.8 × 10^−5^ as the breakage level was changed from 0% to 84%. It is experimentally confirmed that the slope of the imaginary admittance, which is known as a sensor fault indicator in the conventional impedance method [29], can be also used to diagnose the sensor defect for the impedance monitoring via the piezoelectric interface. In summary, an increased value of the slope of the imaginary admittance is an indication for the sensor debonding while a decreased value of the slope is responsible for the sensor breakage, and a stable value of the slope can be interpreted as structural damage but not sensor defects.

**Figure 8:**
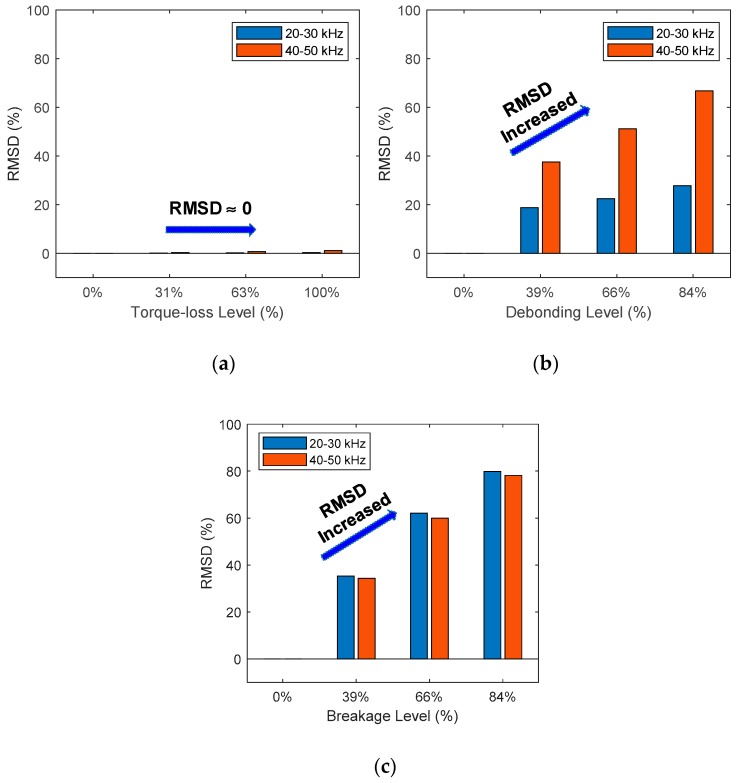
Sensor fault diagnosis using the RMSD metric of non-resonant impedance signatures: (**a**) structural damage; (**b**) sensor debonding; (**c**) sensor breakage.

Next, the sensor debonding and breakage were also diagnosed by using the RMSD metric. It is worthy to note that the structural damage influenced only the imaginary impedance at resonances (see Figure 5b) while the sensor defects caused significant variations in the imaginary impedance over the whole frequency band (see Figure 3b and Figure 4b). Therefore, to differentiate the sensor defects from the structural damages, the non-resonant ranges (i.e., 20–30 kHz, and 40–50 kHz) of the imaginary impedance were selected for the RMSD computation. As observed in Figure 8a, the RMSD metric had no considerable changes when the joint was damaged. In contrast, the RMSD metric was considerably increased as the sensor was debonded or broken, as observed in Figure 8b,c. The results indicated that the debonding or breakage states of the sensor can be diagnosed by using the RMSD metric of the non-resonant impedance signatures.

## 3. Analytical Investigation

The bonding layer plays an important role in the EM impedance formulation to obtain accurate results [29,33,34,38]. The previous impedance models of the piezoelectric interface only consider the effect of the structural damage on the EM impedance and ignored the role of the bonding layer [20,35]. Thus, these models can be used to only assess the structural integrity of the host structure. Herein, we developed a more complete and rigorous impedance model for the PZT interface that can be used for both sensor diagnosis and structural integrity assessment.

### 3.1. A Refined Impedance Model

#### 3.1.1. EM Impedance Formulation

**Figure 9:**
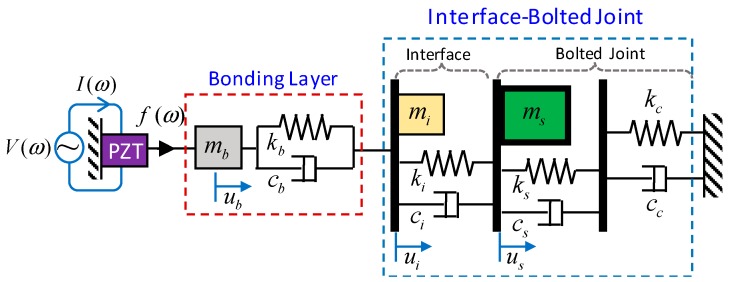
Refined impedance model of the PZT interface-bolted joint system.

As sketched in Figure 9, a refined impedance model considering the bonding layer was newly formulated for the PZT interface-bolted joint system. The refined impedance model is a 3-dof spring-mass-damper system, in which one dof refers to the bolted joint (*m_s_*, *k_s_*, *c_s_*) with the contact parameters (*k_c_*, *c_c_*) representing the bolt preload [39,40], two remaining dofs refer to the interface (*m_s_*, *k_s_*, *c_s_*) and the bonding layer (*m_b_*, *k_b_*, *c_b_*), respectively.

When a harmonic voltage excitation *V* is applied to the PZT, a harmonic force *f_b_* is introduced into the impedance model at the PZT driving point. The equation of motion of the 3-dof system under the external force *f_b_* can be obtained as:

(2) $$\left[\begin{array}{ccc} m_{b} & 0 & 0\\ 0 & m_{i} & 0\\ 0 & 0 & m_{s} \end{array}\right]\left\{\begin{array}{c} {\ddot{x}}_{b}\\ {\ddot{x}}_{i}\\ {\ddot{x}}_{s} \end{array}\right\}+\left[\begin{array}{ccc} c_{b} & -c_{b} & 0\\ -c_{b} & c_{b}+c_{i} & -c_{i}\\ 0 & -c_{i} & c_{i}+{\bar{c}}_{s} \end{array}\right]\left\{\begin{array}{c} {\dot{x}}_{b}\\ {\dot{x}}_{i}\\ {\dot{x}}_{s} \end{array}\right\}+\left[\begin{array}{ccc} k_{b} & -k_{b} & 0\\ -k_{b} & k_{b}+k_{i} & -k_{i}\\ 0 & -k_{i} & k_{i}+{\bar{k}}_{s} \end{array}\right]\left\{\begin{array}{c} x_{b}\\ x_{i}\\ x_{s} \end{array}\right\}=\left\{\begin{array}{c} f_{b}\\ 0\\ 0 \end{array}\right\},$$

where $x_{b},\;{\dot{x}}_{b},\;{\ddot{x}}_{b}$; $x_{i},\;{\dot{x}}_{i},\;{\ddot{x}}_{i}$; $x_{s},\;{\dot{x}}_{s},\;{\ddot{x}}_{s}$ are the displacements, velocities, and accelerations corresponding to the masses *m_b_*, *m_i_*, and *m_s_*, respectively. The terms ${\bar{k}}_{s}=k_{s}k_{c}/(k_{s}+k_{c})\;\mathrm{and}\;{\bar{c}}_{s}=c_{s}c_{c}/(c_{s}+c_{c})$ represent the equivalent stiffness and damping of the bolted joint.

Under the harmonic force $f_{b}=F_{b}e^{j\omega t}$, the steady-state displacements of the system can be defined as:

(3) $$x_{b}=X_{b}e^{j\omega t};\;x_{i}=X_{i}e^{j\omega t};\;x_{s}=X_{s}e^{j\omega t},$$

where *X_b_*, *X_i_*, and *X_s_* are complex vibrational magnitudes of each dof, *ω* is the angular frequency, and *j* is the unit imaginary number (*j*^2^ = −1).

Substituting Equation (3) into Equation (2) will lead to an expression describing the coupled vibration responses of the whole system, as follows:

(4) $$\left[\begin{array}{ccc} -\omega^{2}m_{b}+j\omega c_{b}+k_{b} & -j\omega c_{b}-k_{b} & 0\\ -j\omega c_{b}-k_{b} & -\omega^{2}m_{i}+j\omega (c_{b}+c_{i})+(k_{b}+k_{i}) & -j\omega c_{i}-k_{i}\\ 0 & -j\omega c_{i}-k_{i} & -\omega^{2}m_{s}+j\omega (c_{i}+{\bar{c}}_{s})+(k_{i}+{\bar{k}}_{s}) \end{array}\right]\left\{\begin{array}{c} X_{b}\\ X_{i}\\ X_{s} \end{array}\right\}=\left\{\begin{array}{c} F_{b}\\ 0\\ 0 \end{array}\right\}.$$

The mechanical impedance of the system with the bonding layer $\bar{Z}$ (i.e., the bonding layer-interface-bolted joint system) is defined as the ratio between the excitation force *f_b_* and the velocity ${\dot{x}}_{b}$ at the PZT driving point, as described in Equation (5a). By solving Equation (4), the complex vibrational magnitude of the bonding layer *X_b_* is determined. By substituting *X_b_* into Equation (5a), the mechanical impedance $\bar{Z}$ is obtained, as expressed in Equation (5b):

(5a) $$\bar{Z}=\frac{f_{b}}{{\dot{x}}_{b}}=\frac{F_{b}}{j\omega X_{b}},$$

(5b) $$\bar{Z}=\frac{1}{j\omega }\left(K_{11}-\frac{K_{12}^{2}}{K_{11}+(K_{22}-K_{23}^{2}/K_{33})}\right),$$

where the dynamic stiffness components *K_mn_* (*m*, *n* = 1, 2) are defined as:

(6) $$\begin{array}{l} K_{11}=-\omega^{2}m_{b}+j\omega c_{b}+k_{b};\;K_{12}=-j\omega c_{b}-k_{b}\\ K_{22}=-\omega^{2}m_{i}+j\omega c_{i}+k_{i};\;K_{23}=-j\omega c_{i}-k_{i}\\ K_{33}=-\omega^{2}m_{s}+j\omega (c_{i}+{\bar{c}}_{s})+(k_{i}+{\bar{k}}_{s}) \end{array}$$

In case of the system without the bonding layer (i.e., the interface-bolted joint system), the mechanical impedance ${\bar{Z}}_{o}$ can be obtained from the previous publication [35], as follows:

(7) $${\bar{Z}}_{o}=\frac{1}{j\omega }{\bar{K}}_{o}=\frac{1}{j\omega }\left(K_{22}-K_{23}^{2}/K_{33}\right),$$

where ${\bar{K}}_{o}$ is the dynamic stiffness of the system without the bonding layer.

It is assumed that the mass *m_b_* makes an only ignorable contribution to the dynamic stiffness of the bonding layer $K_{11}$ [32,33]. By substituting Equation (7) into Equation (5b) and neglecting the inertial force of the bonding layer, the relationship between the mechanical impedance with and without considering the bonding layer can be obtained, as follows:

(8) $$\bar{Z}=\frac{1}{1+{\bar{K}}_{o}/K_{11}}{\bar{Z}}_{o}=\gamma {\bar{Z}}_{o},$$

where $\gamma =1/\left(1+{\bar{K}}_{o}/K_{11}\right)$ is defined as the shear-lag index of the bonding layer [23,34], which is controlled by the ratio between the bonding layer’s stiffness $K_{11}$ and the interface-bolted joint system’s stiffness ${\bar{K}}_{o}$.

The overall EM impedance *Z* is a combined function of the mechanical impedance of the system $\bar{Z}$ and that of the PZT sensor *Z_a_* [31,32], as follows:

(9) $$Z(\omega )={\left\{j\omega \frac{\beta w_{a}l_{a}}{t_{a}}\left[{\hat{\varepsilon }}_{33}^{T}-\frac{\gamma {\bar{Z}}_{o}}{\gamma {\bar{Z}}_{o}+Z_{a}}\alpha d_{31}^{2}{\hat{Y}}_{11}^{E}\right]\right\}}^{-1},$$

where ${\hat{Y}}_{11}^{E}=(1+j\eta )Y_{11}^{E}$ is the complex Young’s modulus of the PZT sensor at the zero electric field; $\varepsilon_{33}^{T}$ is the dielectric constant at the zero stress; *d*_31_ is the piezoelectric coupling constant in the *1*-direction at the zero stress; *w_a_*, *l_a_*, and *t_a_* are respectively the effective width, length, and thickness of the sensor; *η* and *δ* are the structural damping loss factor and the dielectric loss factor of the sensor. The wavenumber of the sensor is given as: $k=\omega \sqrt{\rho /Y_{11}^{E}}$, where *ρ* is the mass density of the PZT. The mechanical impedance of the PZT *Z_a_* is computed as $Z_{a}={\hat{Y}}_{11}^{E}w_{a}t_{a}/j\omega l_{a}$. The terms *α* and *β* are the sensor bonding and sensor quality indices, respectively. In the following, the shear-lag, sensor bonding, and sensor quality indices are explained in detail.

#### 3.1.2. Shear-Lag Index

The shear-lag effect is the phenomenon of the difference in the PZT’s strain relative to the host structure’s strain [33]. The shear-lag occurs when the bonding layer is too thick or the shear modulus is too small. In such cases, the force and strain transmission between the PZT and the host structure (i.e., the interface) through the bonding layer is minimal, resulting in erroneous estimation of the EM impedance. In practice, it is recommended to use the bonding layer of high shear modulus and smallest thickness.

As expressed in Equation (8), the mechanical impedance $\bar{Z}$ is decided by the shear-lag index 0≤γ≤1. A decreased value of *γ* indicates an increase of the shear-lag effect. The shear-lag index *γ* = 1 if the dynamic stiffness of the bonding layer is extremely much larger than the dynamic stiffness of the interface-critical joint system (i.e., *K*_11_ ≫ ${\bar{K}}_{o}$). This is an ideal condition (i.e., no shear-lag effect) when the present impedance model will give the same prediction with the previous model [20,35]. In such case, the bonding layer has no influences on the dynamic interaction of the system, and the mechanical impedance $\bar{Z}$ makes the highest contribution to the overall EM impedance *Z*. However, it is almost impossible to have *K*_11_ ≫ ${\bar{K}}_{o}$ in reality. When the shear-lag index *γ* = 0, the mechanical impedance $\bar{Z}$ makes no contribution to the overall EM impedance *Z*. This case happens when the PZT sensor is free (*K*_11_ ≪ ${\bar{K}}_{o}$). Obviously, the proposed impedance model can describe the shear-lag phenomenon for the piezoelectric interface technique and thus provide a more accurate prediction of the EM impedance than the previous model [20,35].

#### 3.1.3. Bonding and Breakage Indices

When the PZT is debonded from the host structure (i.e., the interface), the coupling between the PZT and the bonding layer on the EM impedance measurement is degraded [34]. To simulate the debonding effect of the PZT, thus, a sensor bonding index *α* was added in Equation (9) for the piezoelectric coupling constant *d_31_* of the PZT sensor. It is noted that 0 *≤ α* ≤ 1. A decreased value of *α* indicates a more debonding effect for the PZT. The index *α* = 0 indicates a completely debonded condition of the sensor (i.e., a free PZT condition) and *α* = 1 means an ideal bonding condition (i.e., a perfect bonding condition).

When the sensor is broken/degraded, the effective sizes of the PZT are reduced, causing the variations in the EM impedance according to Equation (9). For the sensor breakage/degradation, therefore, the sensor quality index *β* was introduced into Equation (9) to simulate the reduction in the PZT’s size. It is noted that 0 ≤ *β* ≤ 1. A decreased value of *β* indicates a reduced sensor quality (breakage/degradation). The index *β* = 0 means that the PZT is completely damaged and *β* = 1 indicates an ideally healthy condition of the sensor.

#### 3.1.4. Structural Integrity Index

From Equations (6)–(9), it is clear that the EM impedance is a function of the contact parameters of the bolted joint. When the joint is damaged (i.e., bolt looseness), the contact stiffness *k_c_* of the bolted joint will be altered accordingly [40], resulting in the variation in the measured EM impedance. To represent the effect of the bolt looseness, the equivalent stiffness of the joint can be rearranged, as follows:

(10) $${\bar{k}}_{s}=\xi k_{s},$$

where $\xi =1/(1+k_{s}/k_{c})$ is defined as the structural integrity index, which is decided by the stiffness ratio $k_{s}/k_{c}$. It is noted that 0≤ξ≤1; *ξ* = 1 is an ideal condition when $k_{c}$ ≫ $k_{s}$, indicating that the bolt is perfectly fixed to the joint (i.e., a healthy state); *ξ* = 0 when $k_{s}$ ≫ $k_{c}$, indicating that the bolt in the joint is completely loosened (i.e., bolt looseness). If *k_s_* is unchanged, a decrease of *ξ* is equivalent to a decrease of *k_c_* or an increase of the bolt-loosening severity.

It can be seen from Equations (7)–(10) that the present impedance model (with the bonding layer) obviously results in different EM impedance responses with the previous impedance model (without the bonding layer) [20,35]. The influences of the structural damage, the defects of the sensor, and its bonding layer were taken into account by introducing various parameters into the EM impedance derivation. Hence, the present model can be more rigorous and accurate than the previous impedance model for modeling the EM impedance of the piezoelectric interface-driven system.

### 3.2. Parametric Study

To analytically interpret the influences of the sensor defects on the EM impedance and to demonstrate the theoretical feasibility of the proposed impedance model for sensor self-diagnosis and damage detection, a parametric study was conducted on a simple PZT interface-driven model [20]. The PZT sensor has the following dimensions: *w_a_* = 25 mm, *l_a_* = 25 mm, *t_a_* = 0.51 mm and the following properties: *ρ* = 7750 kg/m^3^, $Y_{11}^{E}$ = 6.098 × 10^10^ N/m^2^, $\varepsilon_{33}^{T}$ = 1.505 × 10^−8^ Farads/m, *d*_31_ = −1.71 × 10^10^ m/V, *δ* = 0.015, *η* = 0.0125. The interface has the following structural properties: *m_i_* = 0.1 kg, *k_i_* = 0.2 × 10^10^ N/m, *c_i_* = 200 N/ms^−1^ and the critical joint has the following properties: *m_s_* = 1 kg, *k_s_* = 2 × 10^10^ N/m, *c_s_* = 200 N/ms^−1^. The contact damping is assumed as *c_c_* = 500 N/ms^−1^.

#### 3.2.1. Shear-Lag Effect

**Figure 10:**
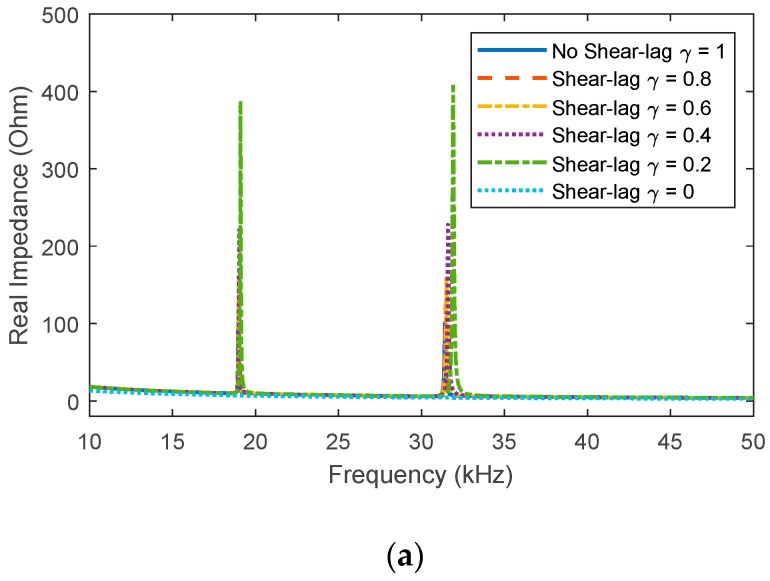

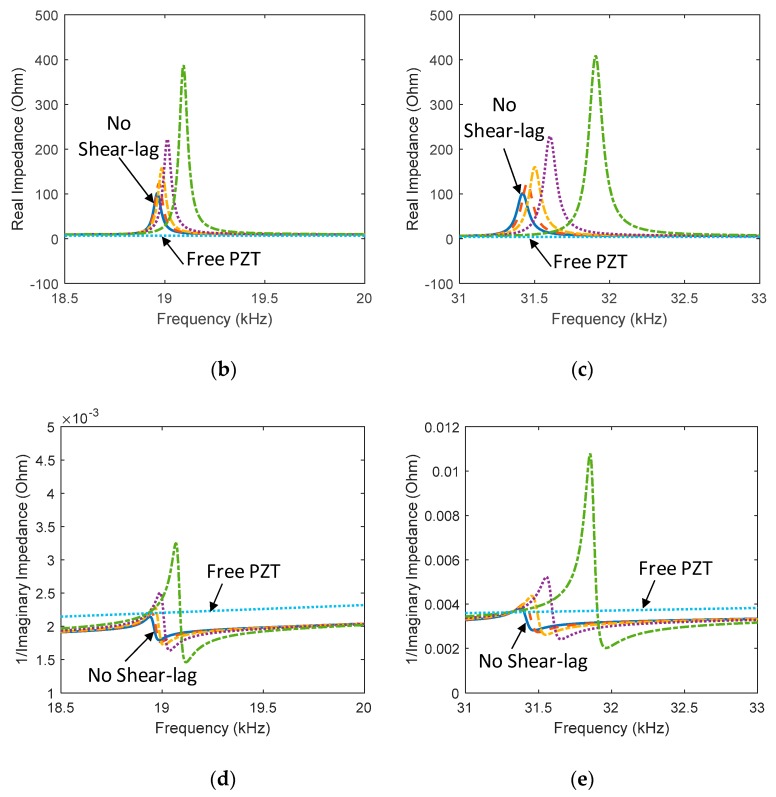
Analytical impedance signatures under the shear-lag effect: (**a**) real part in 10–50 kHz; (**b**) real part in 18.5–20 kHz; (**c**) real part in 31–33 kHz; (**d**) imaginary part in 18.5–20 kHz; (**e**) imaginary part in 31–33 kHz.

At first, the refined impedance model was employed to examine the shear-lag effect between the bonding layer and the interface. Figure 10a shows real impedance signatures in 10–50 kHz corresponding to different values of the shear-lag index (*γ* = 1, 0.8, 0.6, 0.4, 0.2, 0). *γ* = 1 is an ideal case when the bonding layer has no influence on the EM impedance (i.e., no shear-lag effect). *γ* = 0 indicates that the measured impedance will not contain any information about the interface and bolted joint (i.e., a free sensor condition).

Two resonances are observed in the frequency range 10–50 kHz, which are identical to the experimental results in Figure 3a. The two resonant impedance peaks represent the couplings between the PZT, the interface, and the host structure. It is noted that the previous 1-dof model [32,34] can predict only one resonance and unable to predict the EM impedance response for the piezoelectric interface. As seen from Equation (8), the value of *γ* is decided by the stiffness ratio between the bonding layer and the interface-critical joint system. When the dynamic stiffness of the bonding layer is lower, the shear-lag effect becomes more significant, thus resulting in observable changes in the real and imaginary impedances of the two resonances, as zoomed in Figure 10b–e. To obtain more accurate results, the shear-lag effect should not be ignored in the modeling of the EM impedance for the piezoelectric interface.

#### 3.2.2. Effect of Sensor Debonding 

**Figure 11:**
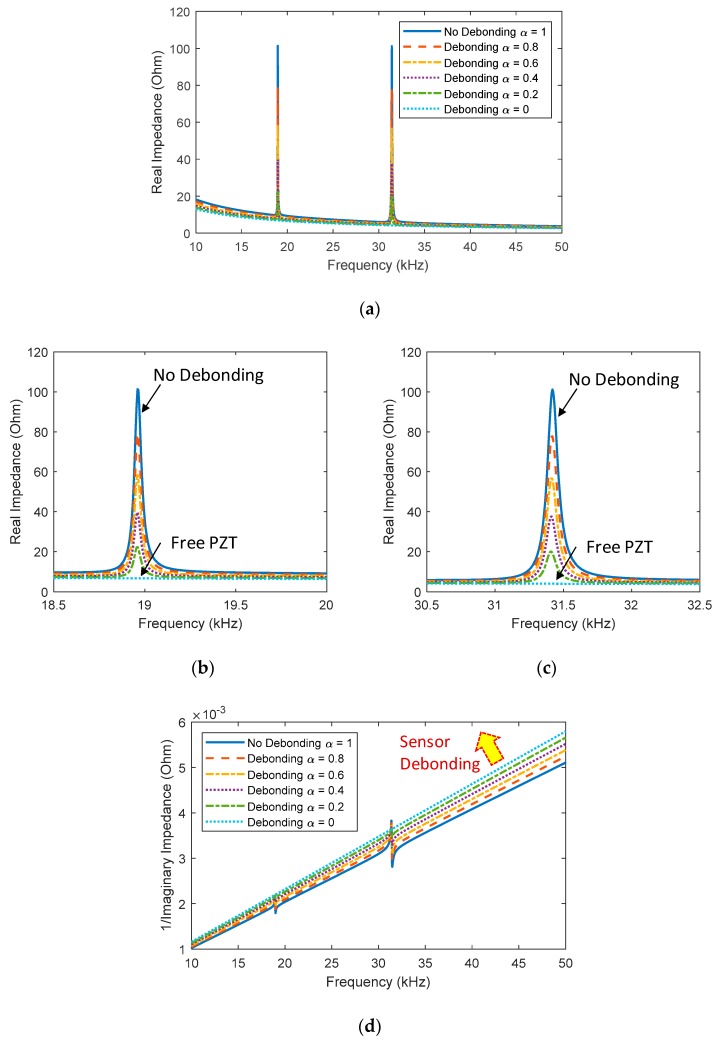
Analytical impedance signatures under the sensor debonding effect: (**a**) real part in 10–50 kHz; (**b**) real part in 18.5–20 kHz; (**c**) real part in 30.5–32.5 kHz; (**d**) imaginary part in 10–50 kHz.

Next, the refined impedance model was also used to analytically estimate the effect of the sensor debonding on the EM impedance for the PZT interface-driven system. Figure 11a shows real impedance signatures in 10–50 kHz corresponding to different values of the sensor bonding index (*α* = 1, 0.8, 0.6, 0.4, 0.2, 0). *α* = 0 means a complete debonding state of the sensor (i.e., a free sensor condition), which is similar to the case *γ* = 0 in Equation (9). *α* = 1 indicates a perfect bonding condition of the PZT. As zoomed in on in Figure 11b,c, the magnitudes of the two resonances were slightly decreased according to the debonding severity of the PZT. The observations from Figure 11d showed that the sensor debonding caused upward shifts in the imaginary admittance. It is analytically confirmed that the sensor debonding occurred in the PZT interface-driven system can be monitored by tracking the changes in the slope of the imaginary admittance.

#### 3.2.3. Effect of Sensor Breakage/Degradation

**Figure 12:**
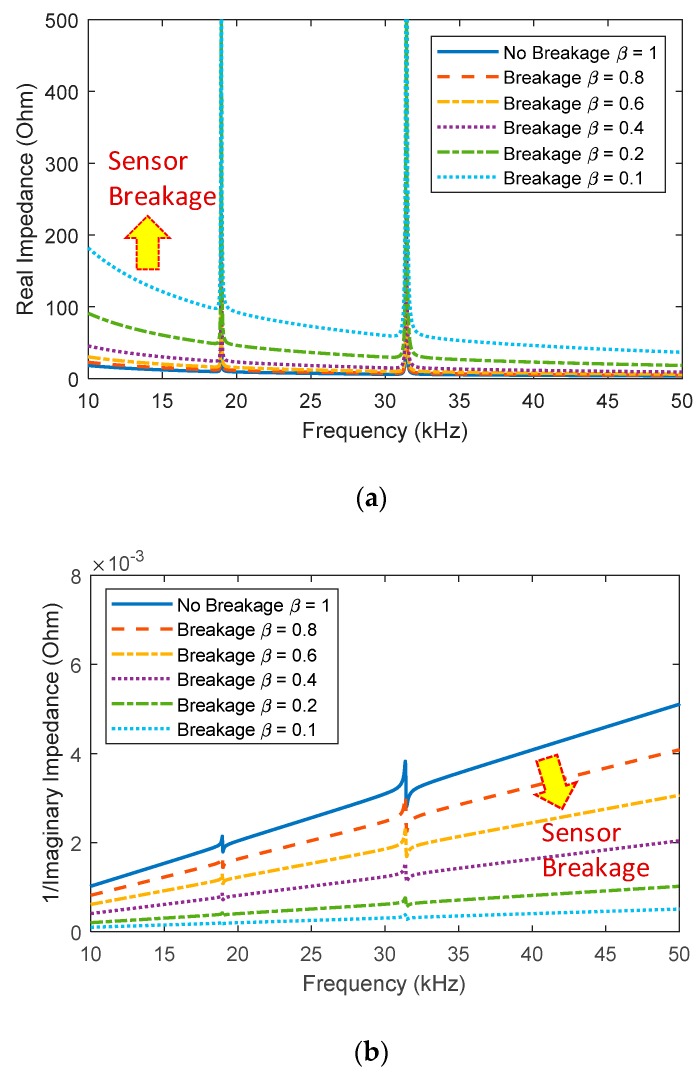
Analytical impedance signatures under the sensor breakage effect: (**a**) real part in 10–50 kHz; (**b**) imaginary part in 10–50 kHz.

The refined impedance model was also used to study the effect of the sensor breakage/degradation on the EM impedance. Figure 12a shows real impedance signatures in 10–50 kHz corresponding to different values of the sensor quality index (*β* = 1, 0.8, 0.6, 0.4, 0.2, 0.1). It is worth noting that a decreased value of *β* indicates a reduced sensor quality (i.e., sensor breakage/degradation) and *β* = 1 means a healthy condition (i.e., no sensor defects). Similar to the experimental results, the analytical model predicted upward shifts in the real impedance and downward shifts in the imaginary admittance according to the sensor breakage/degradation. It is confirmed that the existence of the sensor breakage/degradation in the PZT interface can be identified by monitoring the changes in the slope of the imaginary admittance.

#### 3.2.4. Effect of Structural Damage

**Figure 13:**
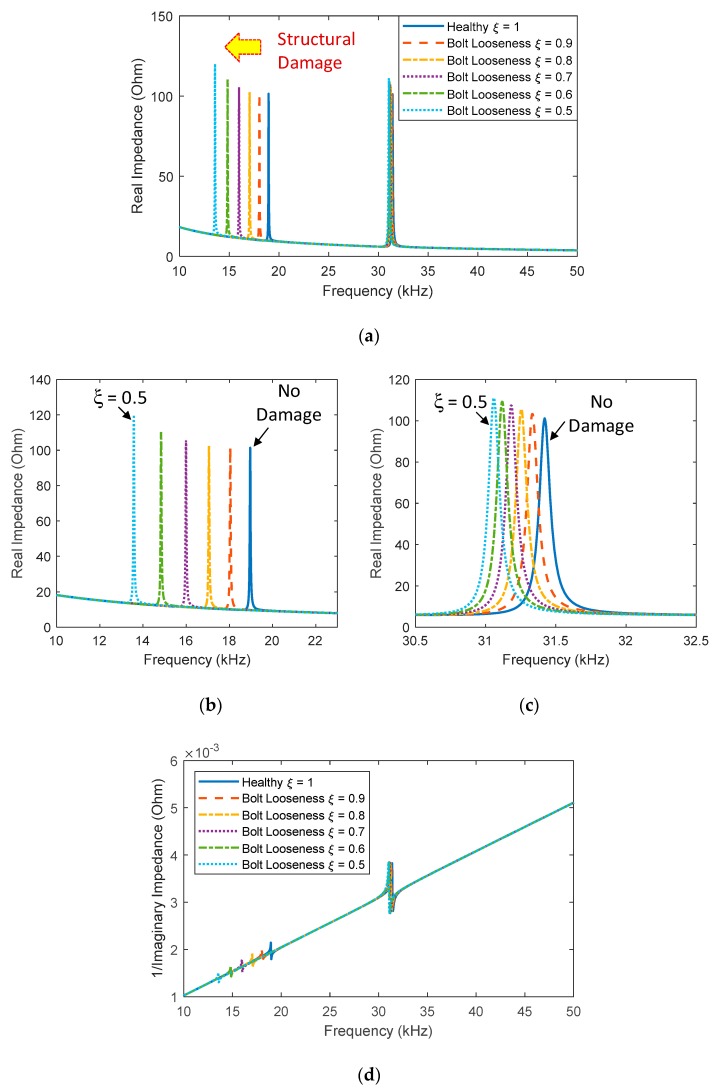
Analytical impedance signatures under the structural damage effect: (**a**) real part in 10–50 kHz; (**b**) real part in 18.5–20 kHz; (**c**) real part in 30.5–32.5 kHz; (**d**) imaginary part in 10–50 kHz.

Finally, the effect of the structural damage (i.e., bolt looseness) on the EM impedance responses was estimated by the refined impedance model. Figure 13a shows the real impedance signatures in 10–50 kHz corresponding to different values of the structural integrity index (*ξ* = 1, 0.9, 0.8, 0.7, 0.6, 0.5). It is noted that a reduced value of *ξ* indicates an increased damage severity and *ξ* = 1 indicates a healthy condition. As seen in Figure 13a, the structural damage caused leftward shifts in the real impedance signatures at the resonances (i.e., decreases in the resonant frequencies). The shifts in two peak frequencies were clearly shown in Figure 13b,c. However, no changes in the slope of the imaginary admittance can be observed under the effect of the structural damage, as shown in Figure 13d. It is confirmed that the structural damage has no influence on the slope of the imaginary admittance, as experimentally observed from Figure 5b.

The analytical results obtained from the parametric study were consistent with the previous experimental observations and thus demonstrated the feasibility of the proposed impedance model for both sensor self-diagnosis and structural damage identification. As compared with an existing impedance model of the piezoelectric interface [20], the proposed impedance model is more complete. Despite its simplicity, the proposed model can accurately estimate the impacts of the sensor defect and the structural damage on the EM impedance responses for the PZT interface-driven system.

It should be noted that the above results were obtained under a constant temperature. The temperature change is able to modify the dynamic properties of the interface and the host structure, as well as the piezoelectric properties of the PZT, consequently causing significant shifts in the measured EM impedance [35]. The temperature effect, therefore, is an important issue that should be properly treated for sensor fault diagnosis in practice. This issue remains for future study.

## 4. Summary and Conclusions

In this study, we experimentally quantified the effects of the sensor defects on impedance monitoring via the piezoelectric-based smart interface and proposed a novel impedance model that can be used for both sensor self-diagnosis and structural integrity assessment. Firstly, an experimental investigation was conducted on a critical connection to analyze the effects of structural damage and sensor defects on the EM impedance. Secondly, a novel impedance model considering the bonding layer was analytically formulated for the PZT interface. To represent the sensor self-diagnosis feature, the shear-lag effect, sensor breakage, and sensor debonding were integrated into the EM impedance formulation. Lastly, a parametric study was performed to verify the feasibility of the novel impedance model.

From the experimental and analytical investigations, at least four concluding remarks can be obtained for impedance monitoring via the PZT interface, as follows:

(1) The structural damage mainly caused the EM impedance variations at resonances while the sensor defects induced the EM impedance changes over both resonant and non-resonant frequency bands.
(2) The sensor debonding caused a decrease in the magnitude of resonances and an increase in the slope of the imaginary admittance. The sensor breakage caused upward shifts in the patterns of the real EM impedance and a decrease in the slope of the imaginary admittance. By contrast, the structural damage did not cause any variations in the slope of the imaginary admittance.
(3) The occurrences of the sensor debonding and breakage can be effectively distinguished from the existence of the structural damage by monitoring the impedance features such as the slope of the imaginary admittance and the RMSD metric of the imaginary impedance at non-resonant ranges.
(4) The refined impedance model can predict more accurate and reliable EM impedance responses for the PZT interface-driven system than the previous 2-dof impedance model. The analytical impedance results were well identical with the experimental observations and thus evidenced the feasibility of the proposed impedance model for sensor diagnosis and structural damage assessment.

This work is expected to provide theoretical and experimental backgrounds for impedance-based SHM practices using the piezoelectric interface, especially with the existence of the sensor defects. Nonetheless, the temperature effect was not considered in the analytical and experimental investigations. In the future, the performance of the developed method will be sufficiently evaluated under temperature variation conditions.
